# Supplementary material for: Inverse-designed large field-of-view polychromatic metalens for tri-color scanning fiber endoscopy
Source: Commun Eng. 2025 Mar 19;4:53. doi: 10.1038/s44172-025-00377-7 (PMC11923133; doi:10.1038/s44172-025-00377-7)
Supplement: Supplementary file 2 — Supplementary Information [file 44172_2025_377_MOESM2_ESM.pdf]

Large field-of-view polychromatic metalens for full-color scanning fiber endoscopy: Supporting information

Ningzhi Xie[a], Zhihao Zhou[a], Johannes E. Fröch[a,b], Matthew D. Carson[c], Arka Majumdar[a,b], Eric J. Seibel[c], Karl F. Böhringer[a,d,e,*]

[a]Department of Electrical and Computer Engineering, University of Washington, Seattle, WA 98195, USA
[b]Department of Physics, Seattle, Washington 98195, USA
[c]Human Photonics Lab, Department of Mechanical Engineering, University of Washington, Seattle, WA 98195, USA
[d]Department of Bioengineering, University of Washington, Seattle, WA 98195, USA
[e]Institute for Nano-engineered Systems, University of Washington, Seattle, WA 98195, USA

Supplementary Note 1: Ray tracing simulation results

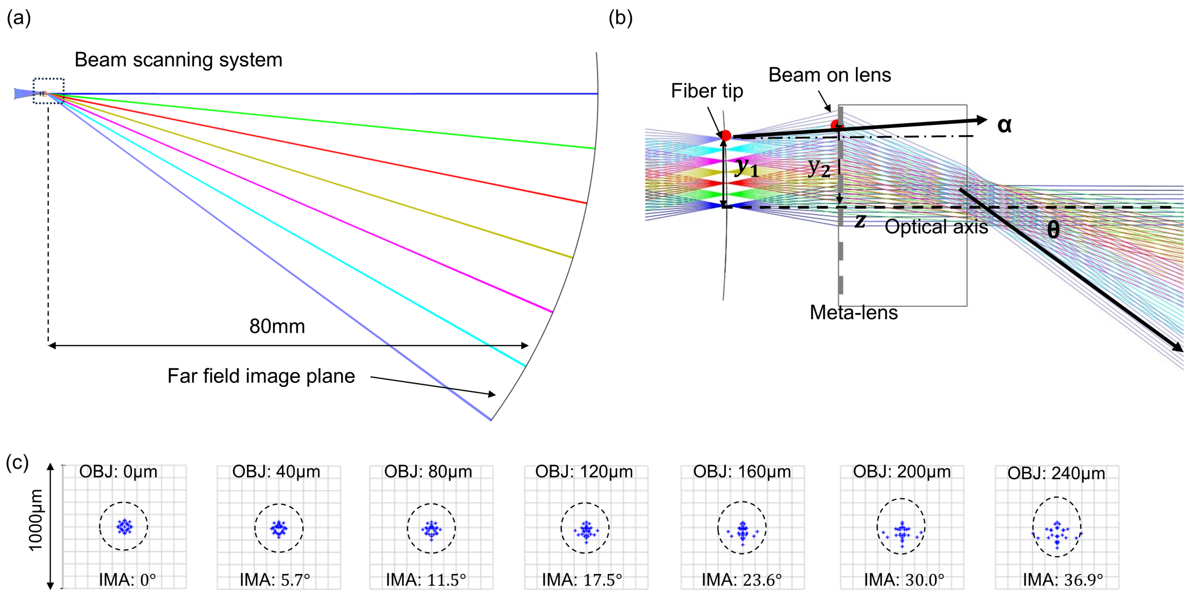

Fig S1 Ray-tracing simulation of the scanning fiber endoscope system. **(a)** Cross-section of the ray tracing simulation of the beam scanning system in SFE. **(b)** Zoom-in cross-section of the scanning fiber and the metalens in the region enclosed by the dashline in (a).**(c)** The spot diagram of the images of 7 point sources on the image plane. The dashed lines indicate the beam spots with a radius of the diffraction limited FWHMs.

beam	1	2	3	4	5	6	7
fiber tip position z along optical axis (μm)	0	-0.16	-0.64	-1.44	-2.56	-4.00	-5.76
fiber tip position y_1 on object plane (μm)	0	40	80	120	160	200	240
beam position y_2 on metalens (μm)	0	44	89	133	177	222	266
beam emitting angle α ($^\circ$)	0	0.29	0.64	0.93	1.22	1.58	1.86
beam steering angle θ ($^\circ$)	0	5.7	11.5	17.5	23.6	30	36.9

Table S1 The position of the point source, the beam position, emitting angles, and the beam steering angles in the ray tracing simulation in Zemax.

The radius of the incident beams on the metalens is 72 μm.

Supplementary Note 2: Simulated beam intensity distribution on the optical plane

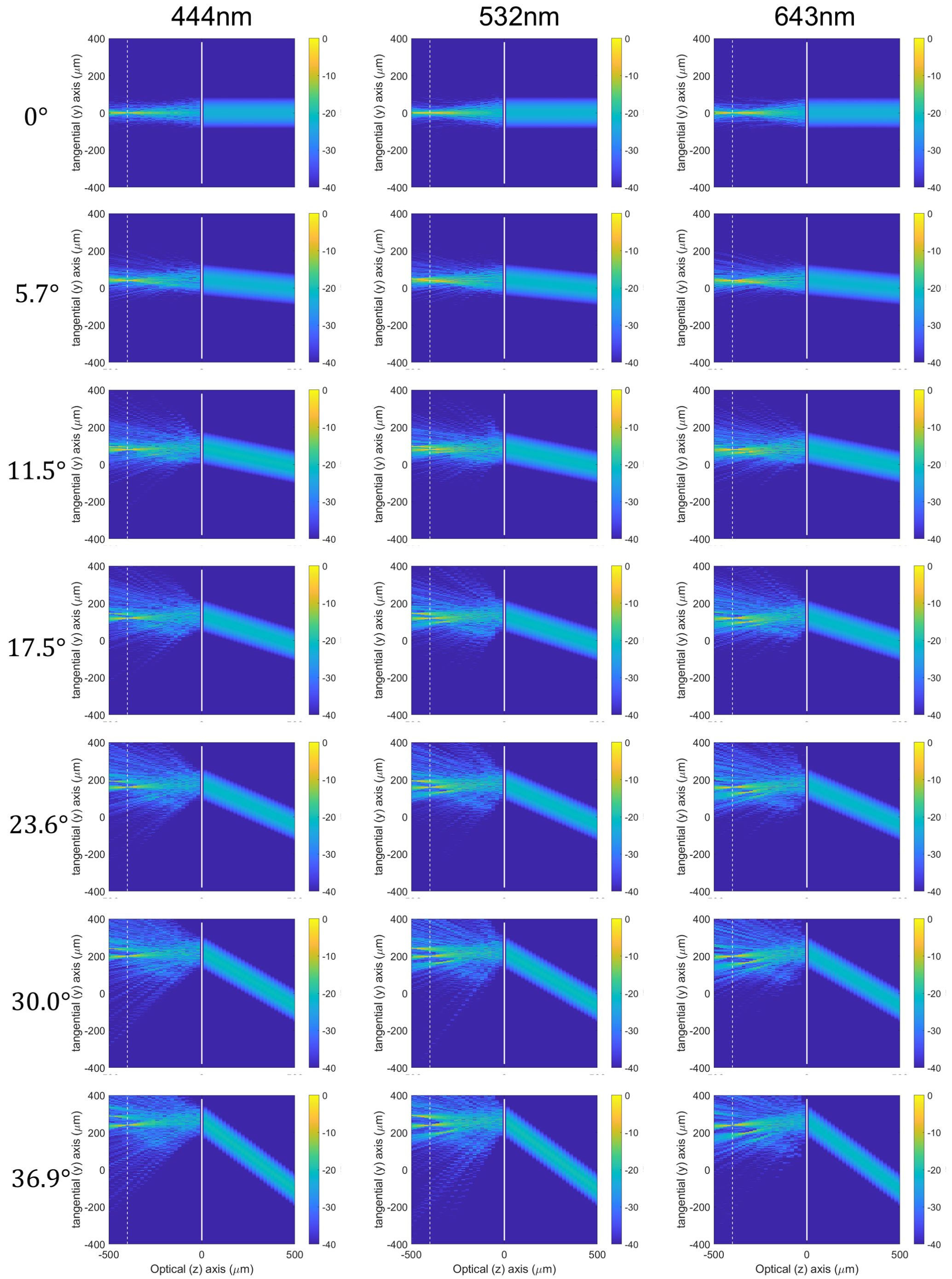

Fig S2 Optical (yz) plane intensity distribution of the beams propagating backward in the inverse design of the metalens. The simulation is performed via angular spectrum method. The beams are incident on the metalens (solid white line) at 7 different angles and positions, which are obtained in the ray-tracing simulation of the system.

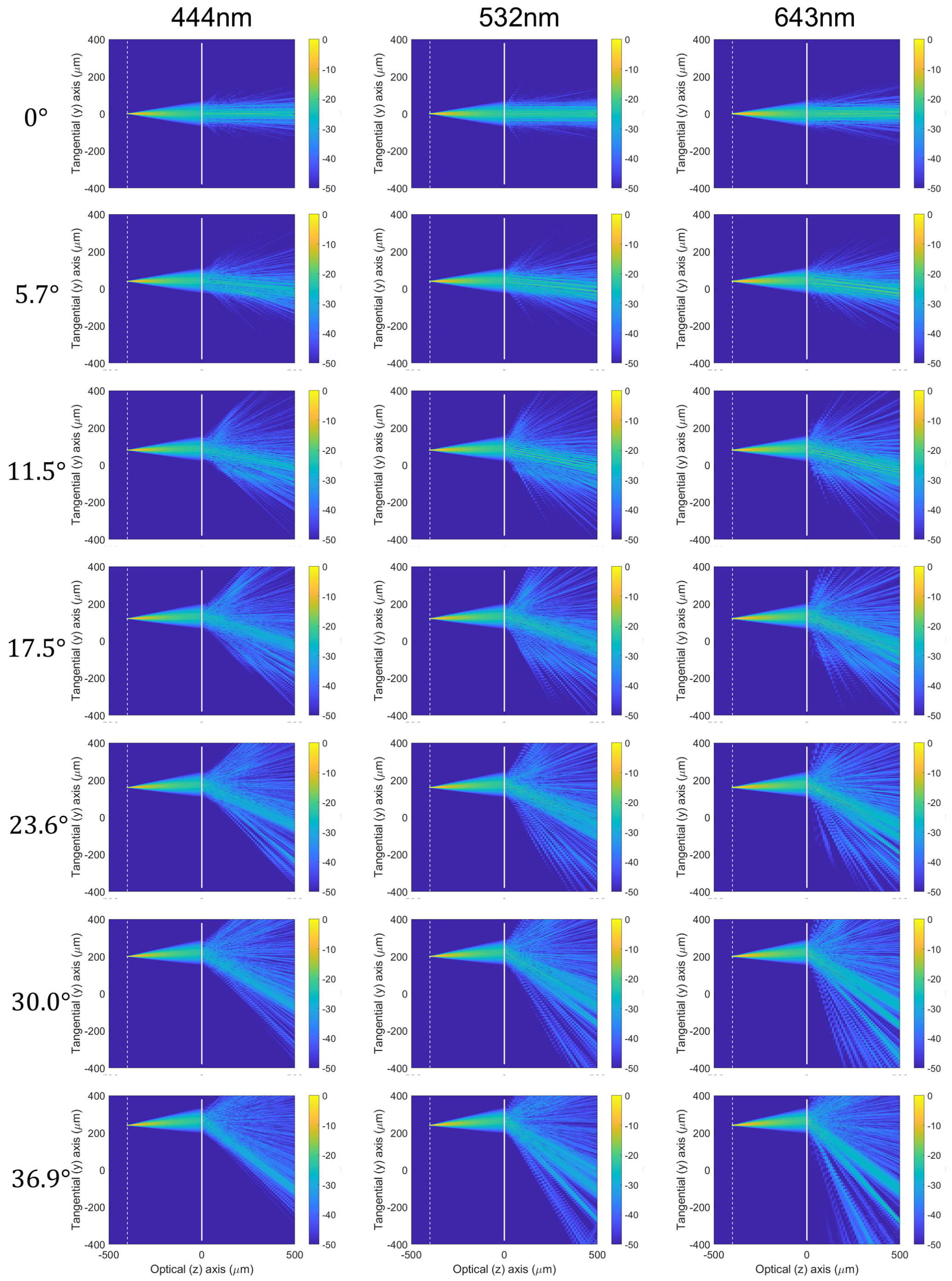

Fig S3 Optical (yz) plane intensity distribution of the beams propagating forward in the functionality authentication of the metalens. The beams emit from fiber tips at 7 different positions on the focal plane (dashed white line).

Supplementary Note 3: The meta-atom library

The target phase profile at a particular radial position, denoted as $\psi(r, \lambda_\mathrm{i})$, can be visualized as a point in a 3D space. The collective target phase profile can be conceptualized as a series of points within a cube in 3D space. If the phase response library of meta-atom covers the entire three-dimensional cube with a side length of 2π, and the transmission of meta-atoms is always 1, it enables independent control over the phase profile at three distinct wavelengths. However, the distribution of points of the phase response and transmission library is constrained by factors such as limitations in refractive index, thickness, and fabrication constraints, thus the efficiency of the lens is lower. Fig. S4 plots the phase response and transmission library of the $750\,\mathrm{nm}$ height Si_3N_4 meta-atoms.

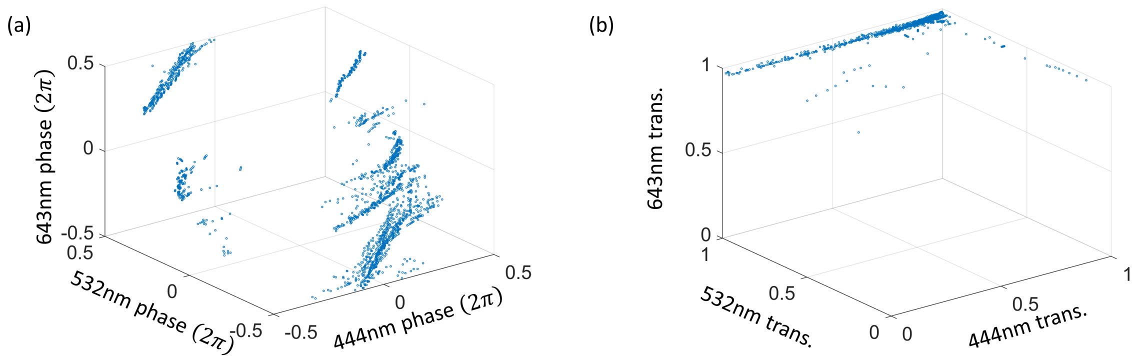

Fig S4 Meta-atom response. (a,b) The phase response (a) and transmission (b) distribution of meta-atoms with $80\,\mathrm{nm} \leq a \leq b \leq 260\,\mathrm{nm}$ at 3 design wavelengths obtained via FDTD simulation.

Supplementary Note 4: Architechture and the training process of the digital neuro network (DNN) meta-model

As can be seen in Fig.S5a, the DNN architecture consists of a 10-layer fully connected network (FCN), with 128 units per layer and a Rectified Linear Unit (ReLU) activation function. The input features of the DNN comprised the incident wavelengths and the two geometric parameters of the meta-atom, while the output consisted of the real and imaginary components of the complex transmission of the meta-atom. In the training process of the DNN meta-model, the dataset included the complex transmission (phase retardation and amplitude modulation) of 5,673 meta-atoms with different geometric parameters simulated using Lumerical FDTD, with 95% allocated for training and 5% for validation. The DNN networks were optimized by minimizing the mean squared error (MSE) between the predicted values and the ground truth (the loss function for the DNN training). This optimization was performed using the Adam optimizer with a learning rate of 10^{-4}, and the model was implemented within the TensorFlow framework. The evolution of the loss function during the DNN training process is shown in Fig.S5b. The computational hardware consisted of a six-core CPU operating at 2.40 GHz, 23 Gigabytes of RAM, and one NVIDIA Tesla V100 GPU. The training process required 13 minutes for 500 iterations, while prediction generation during validation took 27 milliseconds. The validation results of the trained DNN meta-model are plotted in Fig.S5c and d.

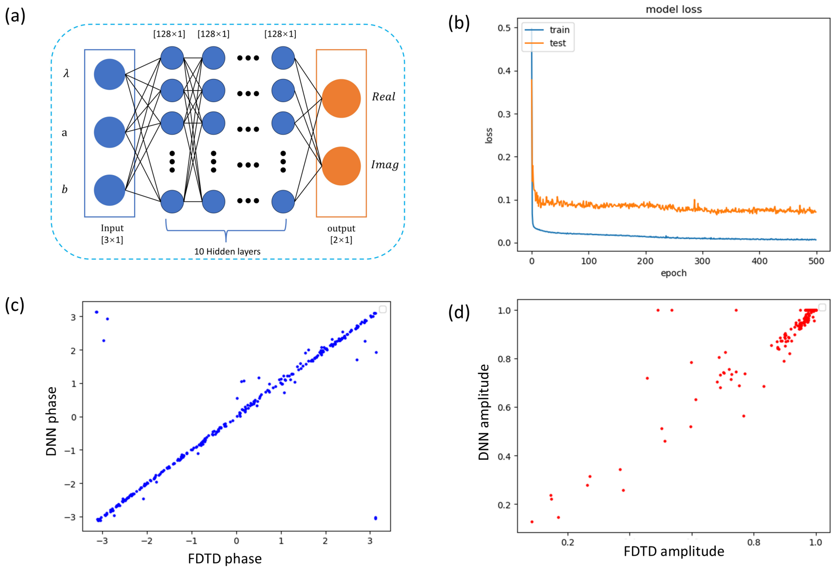

Fig S5 Deep neuro network meta-model for metaatom response prediction. **(a)** The architecture of the DNN meta-model. **(b)** The loss function evaluation during the training process of the DNN. **(c,d)** The phase retardation (c) and amplitude modulation (d) of the meta-atom, DNN predicted values vs. FDTD simulated values (regarded as ground truth) in the validation process of the DNN.

Supplementary Note 5: Calculation of the diffraction limited angular intensity distribution of the steered beam

The diffraction limited angular intensity distribution can be calculated by assuming that the beams after transmitting through the metalens are Gaussian beams with their beam waists positioned at the metalens. The amplitude profiles of these Gaussian beams are assumed to be equal to the incident beams on the metalens:

$$w_0 = r_\mathrm{b} = 40\,\mu\mathrm{m} \tag{S1}$$

where w_0 is the width of the incident Gaussian beam on the metalens, and $2w_0 = 80\,\mu\mathrm{m}$ is the diameter of the incident beam on the metalens, which can be viewed as the effective aperture.

$$w_\mathrm{S} = w_0 \tag{S2}$$

$$w_\mathrm{T} = w_0 \cos\theta \tag{S3}$$

where w_S (w_T) is the sagittal (tangential) Gaussian beam width after transmitting through the metalens.

$$\delta_\mathrm{S} = \lambda/(\pi w_\mathrm{S}) \tag{S4}$$

$$\delta_\mathrm{T} = \lambda/(\pi w_\mathrm{T}) \tag{S5}$$

$$r_\mathrm{S} = \mathrm{FWHM}_\mathrm{S} = \sqrt{2\ln 2}\delta_\mathrm{S} \tag{S6}$$

$$r_\mathrm{T} = \mathrm{FWHM}_\mathrm{T} = \sqrt{2\ln 2}\delta_\mathrm{T} \tag{S7}$$

where δ_S (δ_T) is the sagittal (tangential) diffraction limited diverging angle of the collimated beam, and r_S (r_T) is the sagittal (tangential) diffraction limited beam angular radius.

Supplementary Note 6: Simulated performance of polychromatic metalens with higher efficiency

Meta-atoms with larger height or using higher index materials have a more diverse phase response distributions, as can be seen in Fig.S6. Fig. S7 shows that the metalens using larger height (1500 nm height SiN) or higher index material (750 nm height TiO_2) has a significant higher efficiency than the fabricated metalens in this paper (750 nm height SiN).

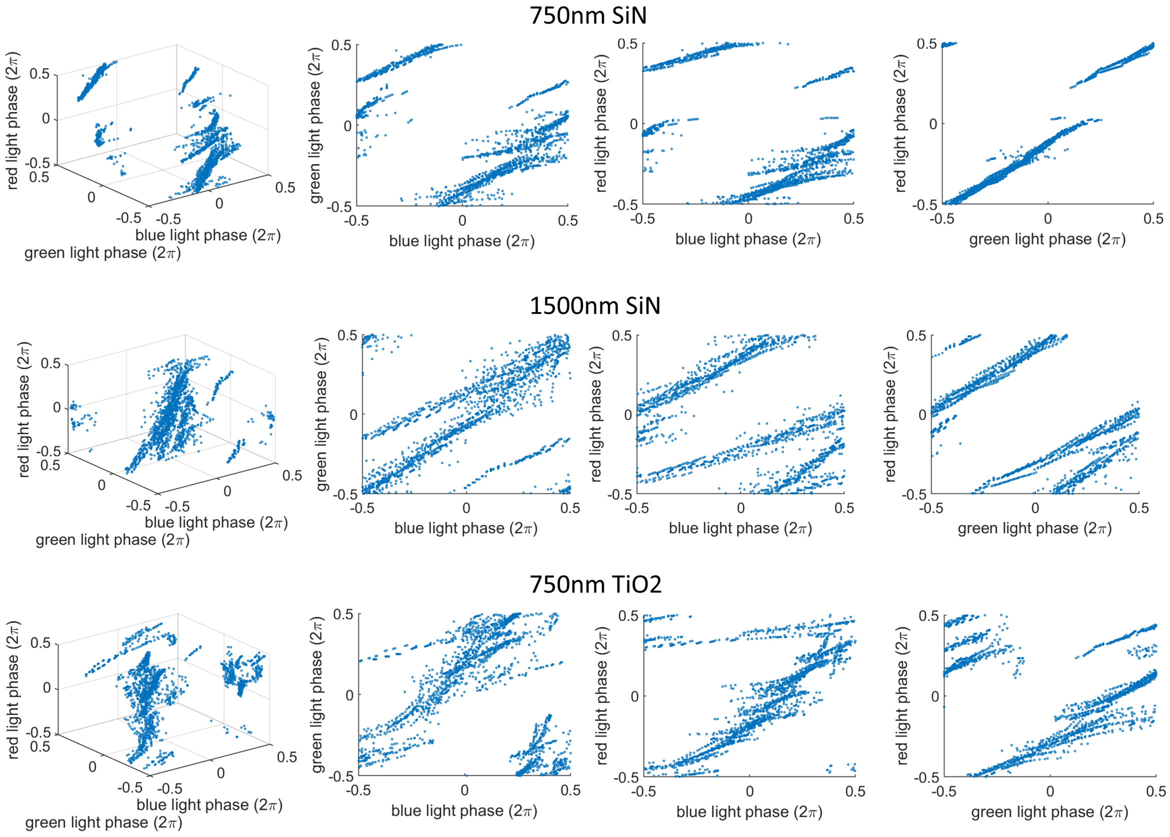

Fig S6 Simulated phase response distribution of meta-atoms at the three design wavelength. **(a)** 750 nm height SiN, **(b)** 1500 nm height SiN, and **(c)** 750 nm height TiO_2

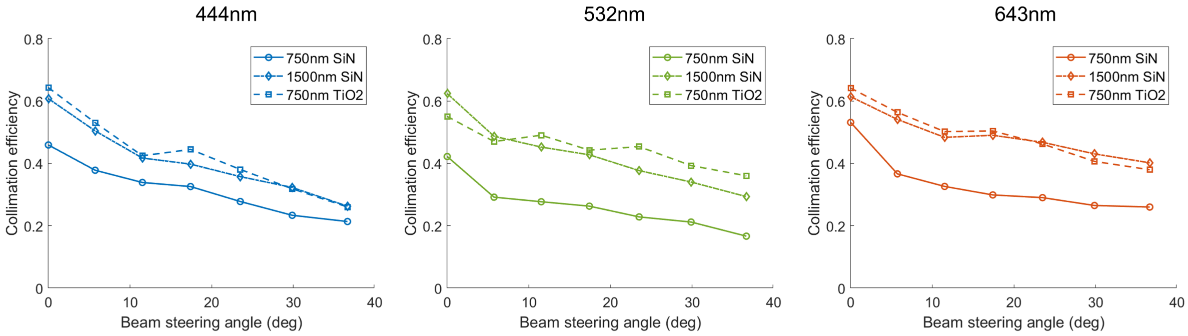

Fig S7 Simulated collimation efficiency of polychromatic metalens consisting of metaatoms with different material and height. The metaatoms are $750\,\mathrm{nm}$ height SiN, $1500\,\mathrm{nm}$ height SiN, and $750\,\mathrm{nm}$ height TiO_2 meta-atoms.

59 ## Supplementary Note 7: Sensor image at multiple wavelengths and steering angles

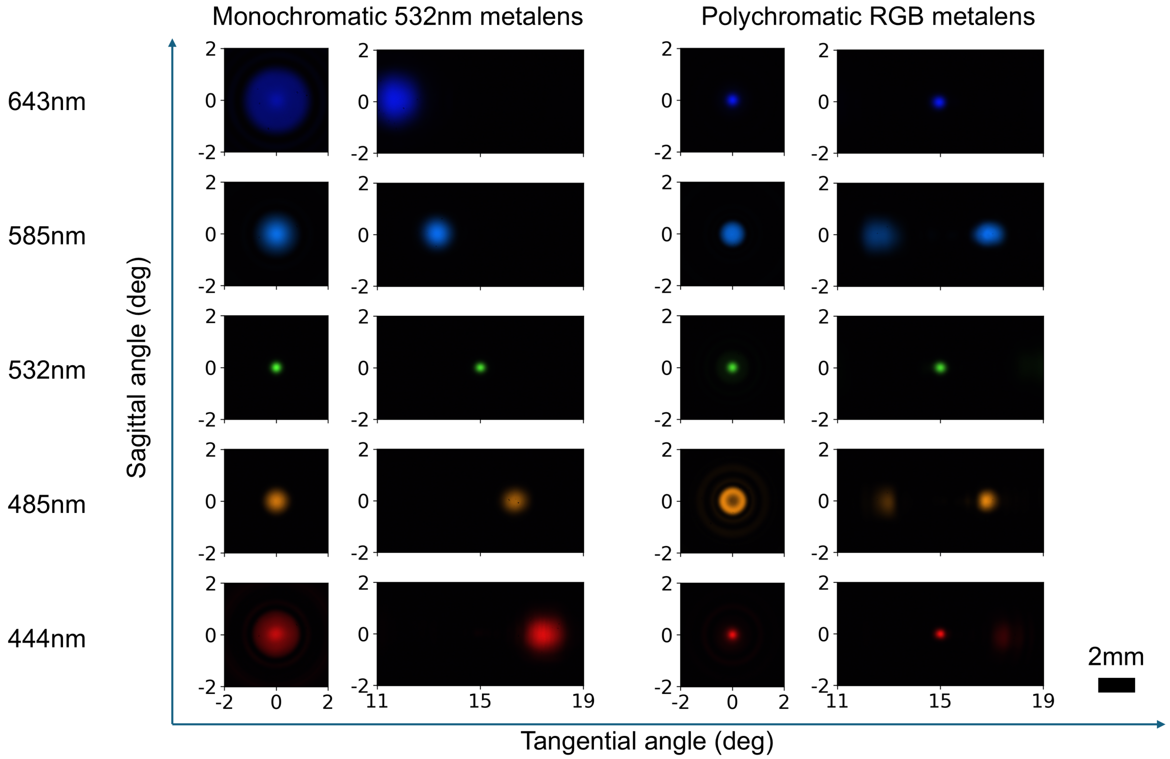

Fig S8 Raw images of the collimation beams captured by the light sensor. The beam is at various wavelengths and the steering angles of $\theta = 0°, 15°$

Supplementary Note 8: Intensity distribution of beams steered to different angles at RGB wavelength

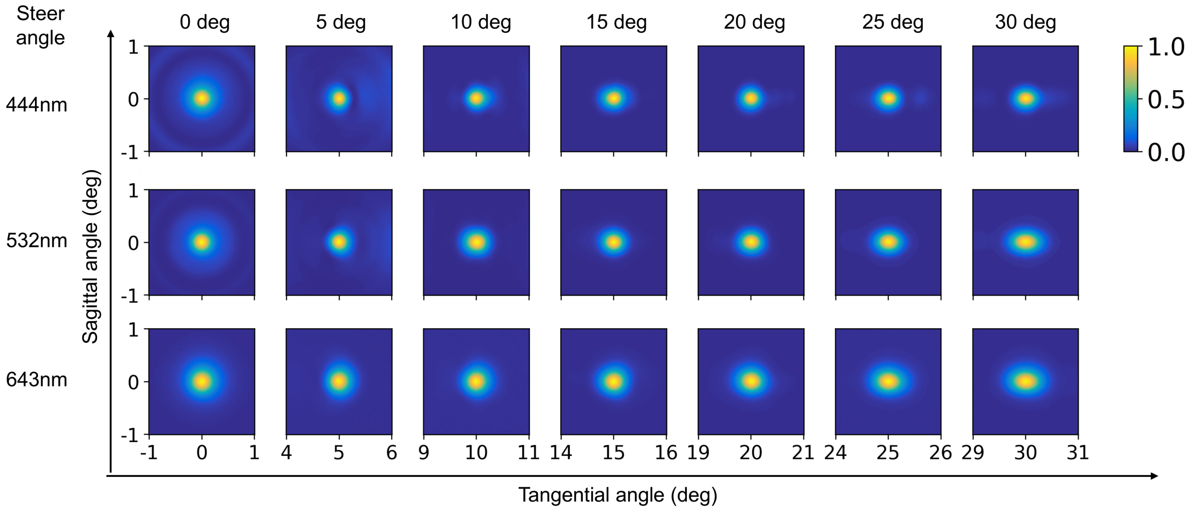

Fig S9 Angular intensity distribution of the beam steered to $\theta = 0$ - $30°$ at the three designed wavelengths.

Supplementary Note 9: Piezo actuator structure and fabrication

The base and the fiber mount are machined then glued to the piezo tube. Then the wires are soldered to the gold-plating on the piezo tube. Finally, the fiber is threaded through and glued into place with epoxy.[1]

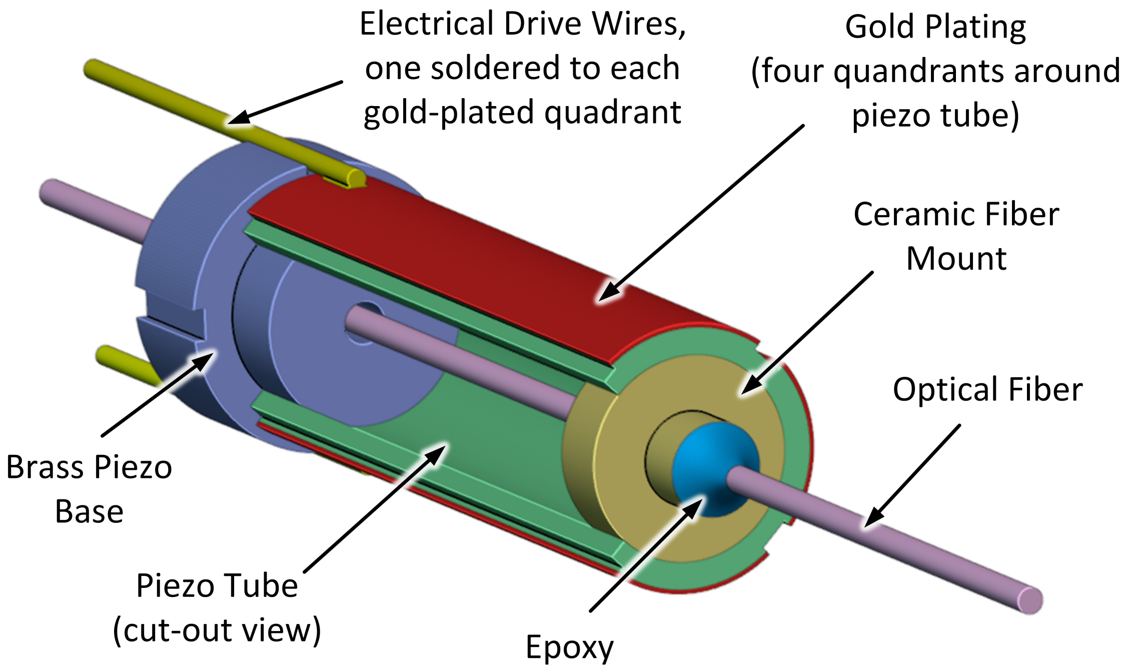

Fig S10 Schematic of the structure of the home made piezo tube actuator for scanning the fiber for the SFE imaging.

Supplementary Note 10: SFE images of a checkboard test pattern

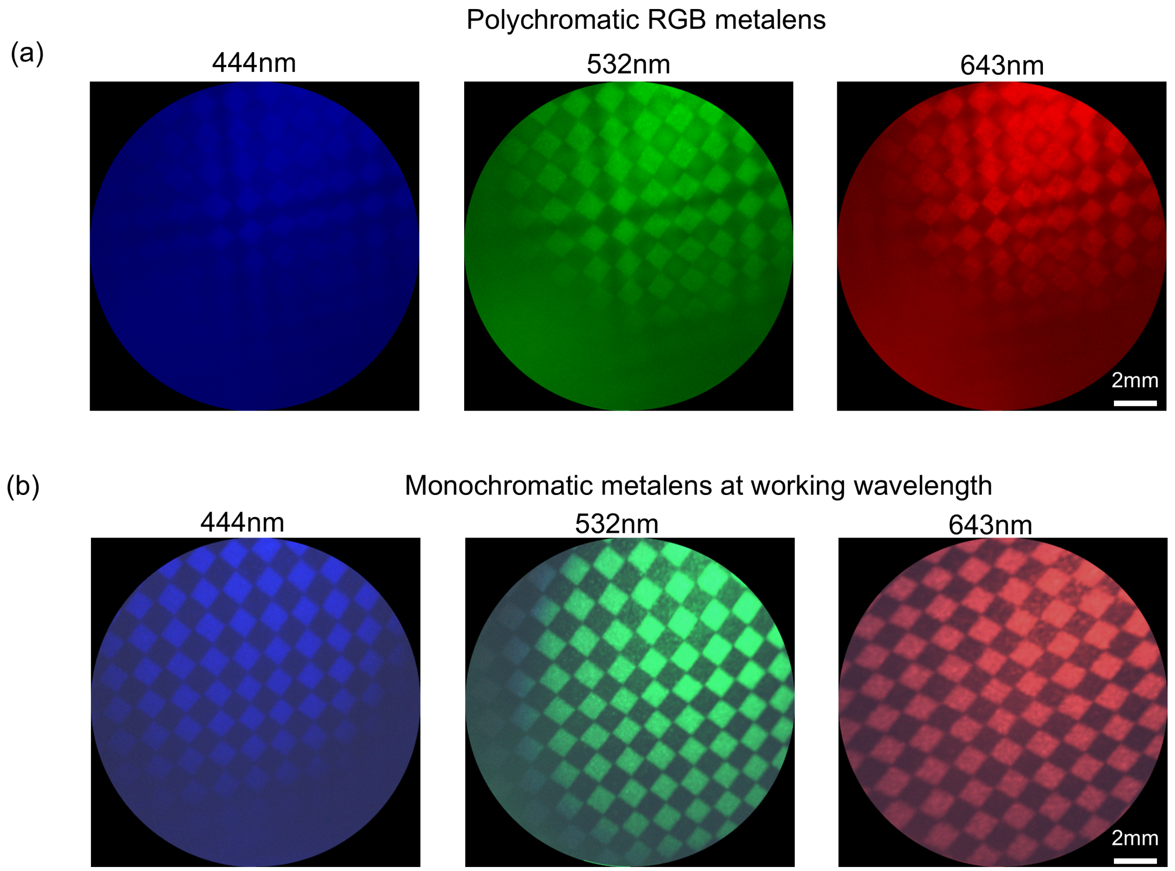

Fig S11 Unprocessed image taken by the SFE platform. The image is taken using the polychromatic RGB metalens at 3 color channels in **(a)**, and 3 monochromatic metalenses designed at corresponding wavelengths in **(b)**. The imaging distance is $14.5\,\mathrm{mm}$, the blocks of the checkerboard have a side length of $1.1\,\mathrm{mm}$, and the angular FOV of this SFE platform is $54°$.

Supplementary Note 11: The comparison of the other polychromatic metalenses and this work

Effective aperture (μm)	Angular resolution (Experiment)	Angular resolution (Diffr. limit)	Angular FOV (Experiment)	Number of wavelengths	On-axis average efficiency	Source
300	0.27° @ 915nm	0.18°		2	$\sim 43\%$	Optica 2016[2]
600	0.16° @ 1300nm	0.13°		3	$\sim 11\%$	Sci. 2015[3]
263	0.37° @ 915nm	0.21°	Near axis	3	$\sim 34\%$	Sci. Rep. 2016[4]
400	0.12° @ 690nm	0.10°		3	$\sim 33\%$	Nano. L. 2018[5]
2000	0.028° @ 658nm	0.019°		3	$\sim 15\%$	Nat.Comm.2022[6]
80	0.41° @ 643nm	0.34°	$\sim 60°$	3	$\sim 32\%$	This work

Table S2 Summary of the performance of various polychromatic metalenses.

For the previously reported polychromatic metalenses, they all acted as focusing lens, where the collimated light were incident on the metalenses, and the point spread function (PSF) at the focal plane were measured. We calculate experimental angular resolutions of these lenses as d_{FWHM}/f, where d_{FWHM} is the full-width-at-half-maximum (FWHM) diameter of the PSF. For our polychromatic metalenses, it primarily acted as a collimation lens, where the light was emitted from a point source at the focal plane, and the PSF was measured at the far field. The Experimental angular resolution is defined as θ_{FWHM}, which is the angular FWHM of the PSF at the far field. The diffraction limited angular resolution for a lens is $1.029\lambda/D_{\mathrm{eff}}$, where λ is the wavelength and D_{eff} is the effective aperture. Our polychromatic metalens has a larger absolute angular resolution, but is still close to the diffraction limit, similar to other prevously reported polychromatic metalenses. Note that for our metalenses, the effective aperture is not the same as the diameter of the metalenses, as only a small portion of the metalens is illuminated by the incident light. This results in a smaller effective aperture and thus a lower angular resolution, but also leads to the large angular FOV that unique to the other polychromatic metalenses. This is because the spatial separation of the lens region that collimates light at different angles effectively reduce the off-axis aberration.

References

1 Rajiv, A., Zhou, Y., Ridge, J., Reinhall, P. G. & Seibel, E. J. Electromechanical model-based design and testing of fiber scanners for endoscopy. *J. Med. Device.* **12**, 041003 (2018).

2 Arbabi, E., Arbabi, A., Kamali, S. M., Horie, Y. & Faraon, A. Multiwavelength polarization-insensitive lenses based on dielectric metasurfaces with meta-molecules. *Optica* **3**, 628–633 (2016).

3 Aieta, F., Kats, M. A., Genevet, P. & Capasso, F. Multiwavelength achromatic metasurfaces by dispersive phase compensation. *Science* **347**, 1342–1345 (2015).

4 Arbabi, E., Arbabi, A., Kamali, S. M., Horie, Y. & Faraon, A. Multiwavelength metasurfaces through spatial multiplexing. *Scientific Reports* **6**, 32803 (2016).

5 Shi, Z. J. *et al.* Single-layer metasurface with controllable multiwavelength functions. *Nano Letters* **18**, 2420–2427 (2018).

6 Li, Z. Y. *et al.* Inverse design enables large-scale high-performance meta-optics reshaping virtual reality. *Nature Communications* **13**, 2409 (2022).
